# How Language Is Embodied in Bilinguals and Children with Specific Language Impairment

**DOI:** 10.3389/fpsyg.2016.01209

**Published:** 2016-08-17

**Authors:** Ashley M. Adams

**Affiliations:** Bilingual Language and Literacy Laboratory, Speech and Hearing Department, Arizona State UniversityTempe, AZ, USA

**Keywords:** embodied cognition, language disorders, bilingualism, motor cortex, action-based language, conceptual representation, clinical practice

## Abstract

This manuscript explores the role of embodied views of language comprehension and production in bilingualism and specific language impairment. Reconceptualizing popular models of bilingual language processing, the embodied theory is first extended to this area. Issues such as semantic grounding in a second language and potential differences between early and late acquisition of a second language are discussed. Predictions are made about how this theory informs novel ways of thinking about teaching a second language. Secondly, the comorbidity of speech, language, and motor impairments and how embodiment theory informs the discussion of the etiology of these impairments is examined. A hypothesis is presented suggesting that what is often referred to as specific language impairment may not be so specific due to widespread subclinical motor deficits in this population. Predictions are made about how weaknesses and instabilities in speech motor control, even at a subclinical level, may disrupt the neural network that connects acoustic input, articulatory motor plans, and semantics. Finally, I make predictions about how this information informs clinical practice for professionals such as speech language pathologists and occupational and physical therapists. These new hypotheses are placed within the larger framework of the body of work pertaining to semantic grounding, action-based language acquisition, and action-perception links that underlie language learning and conceptual grounding.

The debate about whether human perception of the world can be divorced from motor, sensory, and emotional systems is not new. In fact, as far back as Aristotle, philosophers, and scientists were interested in this question (Barsalou, 1999). In the last several decades, the debate continues especially as it relates to speech perception. Some scientists demonstrate clear effects of the motor system on speech comprehension (e.g., Fadiga et al., 2002; Pulvermüller et al., 2003; Pickering and Garrod, 2006), while others argue against a causal role, demonstrating preservation of comprehension skills despite damage to the parts of the brain responsible for speech motor execution (Hickok, 2009; Rogalsky et al., 2011). There is also contention as to conceptual representation. Some argue for a system of abstract and amodal symbols (Newell and Simon, 1972; Pylyshyn, 1973; Fodor, 1975; Dennett, 1981; Haugeland, 1985) and others claim that conceptual representations are grounded in sensorimotor systems (Glenberg, 2011; Kiefer and Pulvermüller, 2012).

While embodied views of language comprehension have gained momentum in the last 20 years, in order to continue to advance the validity of the embodied approach, the theory should demonstrate explanatory power for other kinds of language phenomena such as bilingualism and speech language impairment. The current paper will review the literature on the role of the motor system in language comprehension, and make predictions about how this theory can be extended to bilingualism using popular models of bilingual language processing. Issues such as semantic grounding in a second language and potential differences between early and late acquisition of a second language will be discussed. Next, a review of the literature concerning the comorbidity of speech, language, and motor impairments will demonstrate how embodiment theory informs the discussion of the etiology of these impairments. Finally, based on this discussion, predictions will be made about novel ways to (1) teach a second language and (2) develop interventions for children with speech and language impairments.

## Embodied theories of language comprehension

The major question that the embodied theory of language comprehension attempts to answer is how meaning is represented when understanding language. An understanding of the history of how the theory emerged is important. The late twentieth century gave rise to many amodal theories of language and reading comprehension (e.g., Kintsch's construction-integration model, 1988) that suggested that language comprehension consisted of manipulation of abstract symbols independent of motor and perceptual systems (Kintsch, 1988). Many of these theories were largely inspired by the invention of the computer, and thus promoted a computational view of the brain's role as a language processor. In fact, the advent of latent semantic analysis (LSA) has demonstrated that computational models do have an impressive ability to identify synonyms and engage in tasks (such as essay scoring) that are believed to depend on meaning, given large amounts of input. According to Kintsch and Mangalath (2011), “semantic models like LSA describe what is stored in long-term memory. Long-term semantic word memory is a decontextualized trace that summarizes all the experiences a person has had with that word. This trace is used to construct meaning in working memory” (p. 365). However, even researchers who work with LSA admit “Discourse comprehension requires not only knowledge of what words mean and how they can be combined, but world knowledge beyond the lexical level—knowledge about causal relations, about the physical and social world, which is not captured by our present techniques” (p. 366).

Herein lies the critical difference between amodal and embodied theories of language comprehension. How is it that we “experience” words and what does world knowledge actually consist of? Certainly there is an articulatory and acoustic experience of production and perception of speech, at least when speaking aloud. Pulvermüller et al. (2005) described action and perception links between articulation and acoustic perception based on the principle of Hebbian learning or “what fires together wires together” (Hebb, 1949; Tsumoto, 1992; Artola and Singer, 1993). Principles of Hebbian learning suggest that saying the correct form of the word (and necessarily also hearing it) while in the process of actually performing the action should strengthen the action-perception links that underlie language and ground concepts (Pulvermüller et al., 2006). As Pulvermüller et al. (2006) note, the fibers connecting the auditory and motor regions of the human brain are significantly more well-developed in humans than in apes, providing an evolutionary neurostructural basis for action-perception links. Research has demonstrated such links between activation of motor and sensory brain areas when listening to syllables and words (Zatorre et al., 1992; Pulvermüller et al., 2003; Wilson et al., 2004), and when producing speech (Paus et al., 1996; Watkins and Paus, 2004). This activation in motor areas occurs almost instantly after activation in auditory areas (~20 ms), precluding the alternate argument that activation simply spreads to motor areas after meaning has been processed (Pulvermüller et al., 2003).

Several groups of investigators have explored the causal role that motor systems play in speech perception using transcranial magnetic stimulation (TMS; for a review see Möttönen and Watkins, 2012). Watkins et al. (2003) showed increased motor evoked potentials in lip areas of left motor cortex, but not in hand or homologous right hemisphere lip areas, when watching a video of a person speaking (but not when watching random movements of the same person's face). Fadiga et al. (2002) demonstrated that, when listening to speech, motor evoked potentials in the tongue region were greater when words presented required tongue movement to be produced. This type of effect was shown to increase when the quality of the signal was degraded, either auditorily by embedding sound in noise or visually by increasing the speed of the speaker's articulator movements; these results suggest that motor areas may become increasingly important in situations in which the quality of the signal is less than ideal (Wilson, 2009; Murakami et al., 2011).

D'Ausilio et al. (2009) found that stimulating motor areas responsible for articulation of phonemes facilitated recognition of those phonemes. Schomers et al. (2014) found that differentially stimulating tongue and lip areas of the motor cortex delayed comprehension for words in which the articulator being stimulated was necessary for articulation (i.e., slowed comprehension of *pool* but not *tool* when the lip area was being stimulated). Taken together, this evidence supports the presence of speech mirror neurons; that is, neurons that respond similarly to both the production and comprehension of speech (Galantucci et al., 2006; Guenther et al., 2006). Therefore, there is a wealth of evidence suggesting that there is a causal role of articulatory motor systems in speech perception, especially under degraded conditions, and action-perception links based on Hebbian learning underlie this connection.

The extent to which words are grounded in articulatory and acoustic expression is only one level of grounding. We also experience concepts using our bodies and our senses, yet based on principles of Hebbian learning one would expect the representation of concepts to overlap with motor plans for the production of the word that represents that concept (Glenberg and Gallese, 2012). Take, for example, the word and concept “garlic.” Part of the knowledge of that word is that, in order to produce it, you must begin with a voiced velar stop and end with an unvoiced velar stop. Part of the knowledge is what the word sounds like when it is produced. But, perhaps more importantly, part of that knowledge is what garlic tastes like, its pungent smell, and its shape and color. These characteristics of garlic are experienced via our sensory systems, and embodied theory of language comprehension argues that semantic meaning is stored in these same systems.

There is a large amount of evidence to support the claim that semantic information is stored in a distributed fashion in modality-specific sensory and motor areas. González et al. (2006) showed that reading odor-related words such as *cinnamon* and *jasmine* produced greater activation of olfactory cortex than control words. Similarly, words related to spatial language differentially activate left inferior parietal cortex (Pulvermüller and Fadiga, 2010), superior temporal cortex is differentially active for words related to sound (Kiefer and Pulvermüller, 2012), motor cortex is somatotopically activated when listening to action verbs (Hauk et al., 2004), and sentences describing motion differentially activate parts of the brain responsible for actual visual motion processing (Rueschemeyer et al., 2010). Glenberg et al. (2008) also showed that performing actions that required hand movement away from the body slowed comprehension of sentences describing both literal and abstract motion toward the body. Lesion studies also provide insight into semantic representation in the sense that damage to the above areas differentially affects the ability to process odor, spatial, and motor words respectively (Bak et al., 2001; Kemmerer, 2006; Boulenger et al., 2008; Kemmerer et al., 2012; Trumpp et al., 2013).

There is also evidence suggesting that emotion word meaning is related to the physical expression of those emotions. To investigate the role of the emotional system in language comprehension, Havas et al. (2010) asked adults to read sentences that contained material classified as happy or sad. During the reading of the sentences, muscle activity was measured. The authors hypothesized that the happy sentences would cause greater activation of the facial muscles responsible for smiling and sad sentences would cause greater activation of the facial muscles responsible for frowning. This is exactly what they found. Greater activity of the zygomaticus muscle was found during reading of the happy sentences and greater activity of the corrugator muscles was found during reading of the sad sentences. This suggests that the reading of sentences containing emotional content is directly linked to the parts of the motor system necessary to express that emotion. To follow this up, Havas et al. (2010) used Botox to temporarily paralyze the corrugator muscles (i.e., the muscles necessary to frown) while participants read sentences that described sad and happy situations. Paralyzing these muscles significantly slowed the reading of the sad sentences, while the reading of the happy sentences was unaffected. The fact that paralysis of the muscles necessary to outwardly express an emotion slowed a reader's comprehension of a sentence meant to evoke that emotion suggests that readers may be using their emotional systems during text comprehension.

Zwaan et al. (2002) investigated the activation of the visual perceptual system during sentence comprehension. They asked undergraduate students to read sentences describing either animals or objects differing in shape based on their location (i.e., the eagle is flying vs. the eagle is in its nest). Immediately following presentation of the sentences, they showed pictures to participants that either corresponded with or conflicted with the shape of the object described in the sentence (i.e., an eagle with its wings spread in flight or an eagle with its wings at its side) and asked if the object or animal was present in the sentence. They found that participants were significantly slower at identifying the objects or animals in the pictures when the shape conflicted with that described in the sentence. The slower reaction times in the conflicting condition suggest that the reader had activated their perceptual system during the comprehension of the written material and had to resolve the conflict before comprehension could occur.

Based on the above evidence, researchers in the embodied area have developed a simulation theory of language comprehension (Rizzolatti et al., 1996; Barsalou, 1999; Zwaan, 2004; Glenberg, 2007; Fischer and Zwaan, 2008; Kiefer and Pulvermüller, 2012). According to simulation theory, language comprehension is accomplished by the use of one's own motor, perceptual and emotional systems to simulate the situations described. Glenberg (2011) asserts that comprehension is closely related to bodily abilities and is revealed by the ability to take appropriate action in response to understanding of language or written material. For example, when one hears a sentence such as “He was doing somersaults down the hallway” one would activate the visual perceptual areas necessary to visualize a hallway and real motion, the motor areas necessary to accomplish the action of somersaulting, and the emotional systems that would respond to the unusual sight of a person attempting to somersault down a hallway.

Glenberg and colleagues developed the Indexical Hypothesis to apply simulation theory to language processing (Glenberg and Robertson, 2000; Kaschak and Glenberg, 2000; Glenberg and Gallese, 2012). The hypothesis includes (a) indexing the linguistic symbols (i.e., letters and words) to one's own experience using a perceptual symbol encoded in memory based on neural patterns generated during previous experience with the objects referenced in the text (Barsalou, 1999); (b) deriving affordances from the objects; (c) integrating the affordances based on the syntax (i.e., determining “who does what to whom?” in the sentence). According to Gibson (1977), an affordance is jointly determined by characteristics of physical objects and characteristics of the body. In brief, an affordance is what one can do with the object.

Glenberg and Gallese (2012) also developed the *ABL* model of language acquisition based on Wolpert et al. (2003) *HMOSAIC* more general model of motor control. This model includes a complex set of controllers responsible for generating motor plans, and predictors that make predictions about the sensory consequences of such plans. Central to this model are mirror neuron mechanisms and canonical neurons.

Mirror neurons, first discovered in macaque monkeys by the Parma group in the late 1990's (Gallese et al., 1996; Rizzolatti et al., 1996) and later in humans (Mukamel et al., 2010) are neurons that respond similarly to the execution of an action and to watching that same action performed. Higher-level mirror neurons are theorized to encode the goal of the action (Rochat et al., 2010), while lower level mirror neurons encode the sequence of actions necessary to accomplish that goal. As previously discussed, mirror neurons have been implicated in the role of speech motor processes in language comprehension (i.e., covert activation of mirror mechanisms while listening to speech facilitates speech comprehension; Galantucci et al., 2006; Guenther et al., 2006). Canonical neurons fire in response to object affordances (Glenberg and Gallese, 2012); that is, they fire when completing an action such as grasping, but also when they simply see the object to be grasped. However, canonical neurons do not fire during action observation, differentiating them from mirror neurons.

Gallese et al. (1996) go on to describe how these mirror mechanisms in human Broca's area, which controls both the hands and the speech articulators, represents a form of neural exploitation of this area for action control and for speech. Rauscher et al. (1996) found that restricting hand movements during speech slowed lexical retrieval, further supporting the overlap of these two systems. As such, articulation of action words should prime the motor controllers necessary to take those actions. The reverse should also be true in that performing the action should prime the articulators to say the word that names the action.

Based on the framework of the mirror neuron system, the *ABL* model of language acquisition makes predictions about how children acquire language. Simply put, when young children, at least in Western cultures, are learning language, their more capable language partners tend to label objects in their environment. As such, when children hear words referring to common objects, their speech mirror neurons are activated, and their canonical neurons are activated for the types of actions they can perform with the named object. With repeated exposures, Hebbian learning occurs such that acoustic perceptions, articulatory motor plans, and goal-related motor plans for interacting with the child's environment become linked (Glenberg and Gallese, 2012).

## Embodied theories applied to bilingualism

Taking this evidence base for semantic grounding and theories of language comprehension and acquisition, we will now turn to how these concepts may be applied to bilingualism. But first, why is it important that embodied theory be applied to bilingualism? In most countries in the world, bilingualism is the norm. Some 56% of individuals in 25 European countries reported being bilingual (European Commission, 2006). In the U.S. alone, one of the countries with a lower percentage of bilingual citizens, there are 60.5 million bilinguals (Ryan, 2013), and these numbers are projected to continue to grow at a rapid pace over the next decade (see Shin and Ortman, 2011 for projected population by 2020). Bilingualism extends across all age groups in all levels of society (Grosjean and Li, 2013). However, there is a large amount of within-group variety among bilinguals, making it important to discuss what “bilingual” exactly means. Is someone who has had one class in a second language a bilingual? Does this term apply only to people who learn two languages from birth? Or before a certain “critical period” closes? These questions are important because second language proficiency varies greatly based on factors such as age of acquisition, patterns of language use, similarities among language pairs, language of education, and social standing of the languages in question (Grosjean and Li, 2013). Such factors will need to be taken into account when making predictions about how embodiment of concepts occurs for bilinguals.

**Figure 1:**
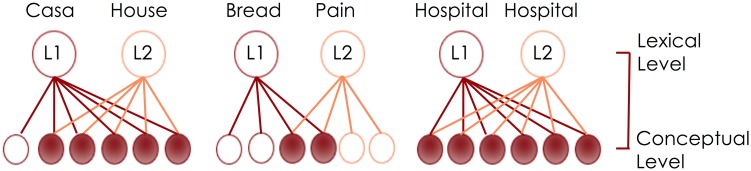
**Adaptation of De Groot's (1992) distributed conceptual feature model**.

Based on Pulvermüller's research on semantic grounding and Glenberg and Gallese's *ABL* model, predictions can be made about how this process might occur in early simultaneous bilinguals. Children who acquire two languages from birth learn language in a very similar way to monolingual children. As they interact with their parents, objects are named for them in one or both of their languages depending on language practices in the home. If there are not cultural differences in appropriate ways to interact with an object, children may simply have an additional controller module that contains the speech motor commands for producing a word in two languages. Similarly, to the extent that there are not expected differences in sensorimotor experience of concepts (e.g., apple and manzana), the only additional component to be expected for semantic grounding is a link between the articulatory motor plan for a word in the second language and the meaning of that word as it is grounded in sensorimotor experience. However, children who are bicultural bilinguals may need separate conceptual representations for common objects or actions in their environment. Consider the concept of bread, for example. For a speaker of English in the United States, this would likely evoke the image of a loaf of sliced white or wheat bread that you buy in a grocery store. However, for a French-English bilingual/bicultural child, *pain* might evoke a different image, one of a baguette that you buy from the *boulangerie*. De Groot's (1992) distributed conceptual feature model suggests that bilinguals' conceptual representations may have overlapping and non-overlapping features as seen in Figure 1 below.

While De Groot conceptualized these semantic features as abstract and amodal, this same idea could be reconceptualized in terms of semantic grounding. To continue with the bread example, the English and French concept would differ in terms of visual and likely to an extent in gustatory information, also likely in some affordances (i.e., grabbing a piece of sliced bread vs. a baguette would require a different set of motor commands) but possibly not in olfactory information. Therefore, to the extent that sensorimotor information overlaps between the two concepts, one would expect the neural network responsible for semantic grounding to be the same. However, differences in sensorimotor experiences related to the two different concepts would result in neural networks that grounded the concepts in different sensorimotor information.

Jared et al. (2013) tested the hypothesis that there may be differential culture- and language-specific activation of concepts and found that participants were faster to identify culturally biased items (e.g., a traditional Chinese mask) when they read the word *mask* in Chinese rather than in English. From the perspective of simulation theory, this advantage in reaction time occurred because hearing the word *mask* in Chinese activated a specific perceptual memory that was specific to the participant's experience with a mask in their Chinese language environment. The background knowledge that was activated is not composed of arbitrary linguistic information, but instead includes activation of the motor, sensory, and perceptual system. If two languages are learned in different environments at different times, there may be differences in the knowledge that is activated depending on the language of input.

**Figure 2:**
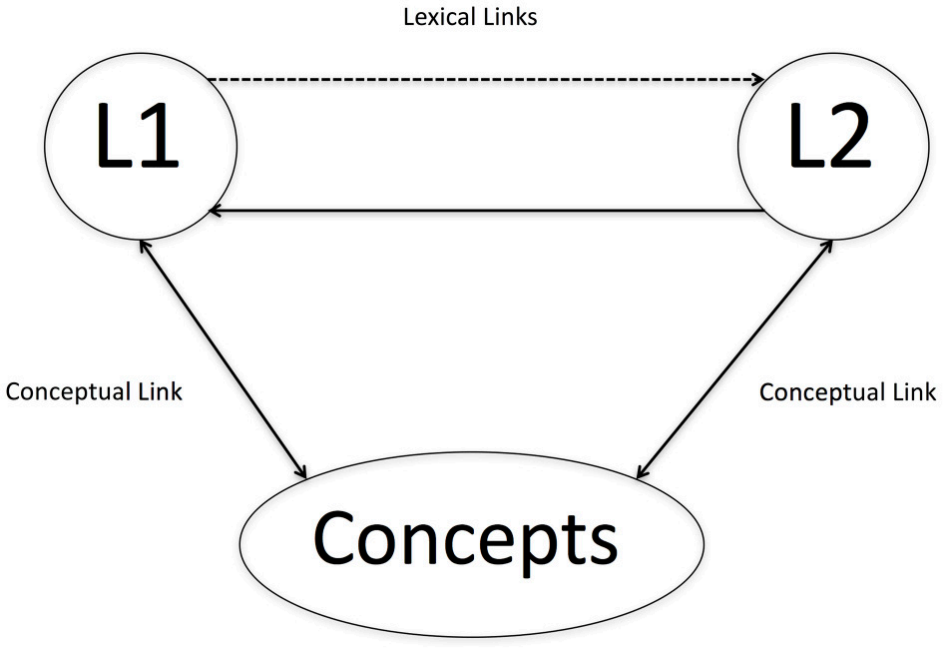
**Adaptation of Kroll and Stewart's (1994) revised hierarchical model**.

Making these kinds of predictions becomes more complicated when a language learner has acquired a second language later in life, especially if the primary modality in which that language was learned was in a classroom. In this case, a new hypothesis generated from the review of literature concerning embodied language is that late second language learning may be critically different from first language learning in the sense that it may be less likely that vocabulary acquisition happens alongside actual sensorimotor experience (Dudschig et al., 2014). In the case that the learner has a grounded conceptual representation already in place, he or she may simply link the new phonological representation to the previously grounded concept. Kroll and Stewart's (1994) revised hierarchical model, seen below in Figure 2, makes just such a prediction.

When a second language is still emerging, according to this model, the first language mediates L2 access to the conceptual store. As L2 proficiency develops, necessarily through experience with the language, a semantic link begins to strengthen between L2 and the “conceptual store,” such that eventually first language mediation may not be necessary if a high enough level of proficiency is achieved. Or, to make a new prediction, one might hypothesize that as new sensorimotor experiences take place in the second language environment, the grounded conceptual representation may gradually shift to incorporate these new experiences. One could conceptualize the arrows in the figure above as neural networks of sensorimotor information that ground concepts. Certainly this is a testable hypothesis and by giving a novice second language learner new experiences with an object, action, or feeling, it should be possible to measure differential activation as it relates to the quantity and quality of sensorimotor experience.

In the case that the learner has no concept in place to attach to a new lexical item, an entirely new, grounded representation must be created. Take for example the distinction of *querer* and *amar* in Spanish; the former refers to non-romantic love and the latter to romantic love. This distinction does not exist in English, although certainly the emotions that platonic and romantic loves generate are different. Therefore, the second language learner of Spanish may gradually ground the word *amar* in feelings of romantic love and the word *querer* in the feelings of familial love, given enough experience with the language.

An alternative framework in which to consider bilingual language processing within an embodied framework is the Bilingual Interactive Activation Plus Theory (BIA+, Dijkstra and van Heuven, 2002). According to this model, representations are stored in an integrated way, regardless of the language to which they belong. However, there are different resting-level activations connecting lexical items with the semantic system, based on proficiency and word frequency. Therefore, a bilingual who is less proficient in their L2, for example, would require greater activation for the neural network responsible for a given concept to “fire.” Words that are less frequent or more unfamiliar to the speaker would have resting-level activations that were more negative, and would therefore require greater activation. One could apply knowledge of both embodied language theories and the BIA+ model to extend current knowledge and conceptualize that embodied neural networks of sensorimotor experience may have differential resting-level activations that link the speech motor, acoustic, and semantic information for a given word according to factors such as proficiency, word frequency, and amount of sensorimotor experience with a given object, action, emotion, etc.

In the last 5 years, investigators have started to explore how embodied/simulation processes occur in both languages of a bilingual. Vukovic and Williams (2014) had 24 highly proficient Dutch-English speakers listen to sentences that implied distance (e.g., the microphone across the room vs. the microphone in your hand) in their L2 (English). Critically, target sentences contained an interlingual homophone (a word that sounds the same but has different meanings in the two languages) to test whether simulation of distance occurred in both languages simultaneously. Participants heard a sentence like “on the plate in front of you is a bone.” Therefore, participants should be faster to identify a large or close-up picture of a bone than a smaller picture (Winter and Bergen, 2012). However, *bone* in Dutch means *beans*. Vukovic and Williams set out to determine if, after hearing the sentence above, participants would be slower to reject a large close-up picture of beans rather than a smaller picture. This would only be true if simulation of distance was taking place in both languages simultaneously. Indeed they found just this effect. The results of this study coincide with previous work suggesting simultaneous activation of both languages in bilingual language processing (Lagrou et al., 2011), and also speak to the automatic process of simulation, even when meaning is only subconsciously processed.

Another study with highly proficient German-Dutch bilinguals used fMRI to study L2 embodiment of action verbs and demonstrated increased activation of the motor and somatosensory regions when listening to simple verbs in their L2 (De Grauwe et al., 2014). Some of the verbs presented were cognate words (similar phonologically and orthographically to German verbs with the same meaning) and the authors found no significant interaction between cognate status and motor activation, suggesting that embodiment of action verbs in L2 is not mediated by first language embodied concepts, at least for highly proficient bilinguals.

The proficiency in the second language may play an important role in how concepts are embodied. In another study with later (and presumably less proficient) second language learners, Dudschig et al. (2014) presented German-English bilinguals with variations of the Stroop task to test for second language (L2) simulation effects. In the first experiment, participants were presented with words that implied a typical location (e.g., bird, shoe, airplane, root) in English, their L2. These words were presented on a screen and were written in one of two colors. Participants were asked to respond to one color with a movement that required hand movement up in space and to the other color with a movement requiring downward motion. The participants were instructed to ignore the meaning of the words. Results showed a significant word-direction and response-direction interaction, such that participants were faster to correctly identify the color by moving their hand upwards when the location implied by the word they read in their L2 was also upwards. Interestingly, similar results were found using emotionally charged words based on previous work by Meier and Robinson (2004) that suggested that positive-valence emotion words are identified faster when they are presented in the upper half vs. the lower half of the screen, and vice versa for negative-valence words. Dudschig et al. (2014) corroborated these results by again finding a word-direction and response-direction interaction for emotion words. These results suggest that semantic grounding in sensorimotor experience occurs in similar ways for both the first and second languages, even when the second language is acquired later in life.

However, another study investigated further the role of proficiency in embodiment of a second language. Bergen et al. (2010) showed adult second language learners two pictures, one of a written verb (e.g., run) and then a drawing of a person doing an action that was either accomplished with the same effector as the verb seen (e.g., kick) or a different effector (e.g., punch). Participants were slower to reject drawings that showed actions accomplished with the same effector implicated by the verb they read, which the authors theorized was due to interference of simulation of the original action. Importantly, accuracy of performance on the task, a proxy for language proficiency, was related to this interference effect; that is, more proficient second language learners had a greater “native-like” interference effect. However, a test of receptive vocabulary was not significantly related to the effect, indicating a complex interplay between language proficiency and the extent to which language can be embodied. Harris et al. (2003) demonstrated that, for late second language learners, taboo and negative words elicited higher levels of skin conductance in the first language relative to the second language, suggesting that embodiment effects may differ between languages of varying proficiency. More work is necessary to determine the extent to which concepts are embodied in more and less proficient speakers of a second language.

A consideration of the totality of this evidence allows for predictions about effective second language teaching and further development of the current theory of embodiment. In very simple terms, second language learning, much like first language learning, should take place in authentic environments and should involve discussion of the immediate perceptual environment, accompanied by interaction with that environment. According to Macedonia (2014), “embodiment is giving language education a cutting edge by authorizing it to consider the body as a learning tool” (p. 4). Several studies have demonstrated that gestures can improve memory for new words and phrases in a second language (Macedonia and Knösche, 2011; Bergmann and Macedonia, 2013; Porter, 2016). Mayer et al. (2015) provided neurological evidence of this improvement. They taught adults foreign language words and their translations and either had participants perform a gesture related to each word, trace a picture related to each word, or simply learn the word verbally (much like what happens in the classroom). They found that the group that received the training with self-performed gestures recalled more words correctly than the group that learned in the verbal only modality; this behavioral finding was directly related with the amount of activity in the left motor cortex and the right biological motion-sensitive superior temporal sulcus (Mayer et al., 2015). The results of this study not only provide strong direct evidence for semantic grounding of words in a second language, but also speak to potential strategies that may be effective for second language teaching.

Atkinson (2010) advocates for a sociocognitive approach to second language teaching that encourages the use of gestures, body positioning, and what he terms “alignment” to improve memory for new words and concepts. A study that followed university students as they learned a second language found that, in contrast to classroom learning, learners' bodies demonstrated a higher level of engagement during a conversational exchange in the second language (Tanous, 2014). This demonstrates that when communication partners are exchanging meaningful information (but not necessarily when they are being taught in a classroom environment) their bodies reflect this language learning experience. As such, teaching of a second language may be reconsidered as guided discovery of the world in a new language environment instead of conveying abstract knowledge in a lecture format (Atkinson, 2010).

In summary, there is a wealth of evidence demonstrating the role of the motor system in language comprehension. The discovery of mirror neurons in human cortex has revived the discussion of the causal role motor neurons play in language processing. Relatedly, sensorimotor experience of the world leads to grounding of semantic information using the brain's sensory, motor, and emotional systems. Simulation theory suggests that concepts are represented using these same systems, as opposed to in some abstract, amodal conceptual store. Emerging research demonstrates that embodiment processes occur in a similar way for highly proficient bilinguals, but may differ in some ways for less proficient second language learners. Several popular models of bilingual language processing allow for predictions about how and why these differences occur. Future research that explores the connections between a well-defined construct of language proficiency and embodiment of concepts in a second language will help to illuminate this issue. Finally, the principles of embodied cognition and simulation theory provide exciting insights into new ways to teach learners a second language. A randomized controlled experiment that explores using the “guided discovery of the environment” method of second language teaching vs. traditional classroom teaching would be one interesting way to test the hypothesis of how embodiment theory applies to second language learning.

## Embodied theories applied to specific language impairment (SLI)

Specific language impairments affect ~7–10% of the population (Tomblin et al., 1997). According to the National Institute on Deafness and Communication Disorders, specific language impairment is “a language disorder that delays the mastery of language skills in children who have no hearing loss or other developmental delays.” Decades of research have established that the primary difficulties for children with SLI are in phonological processing (Weismer et al., 2000; Botting and Conti-Ramsden, 2001; Graf-Estes et al., 2007), morphology (Rice and Wexler, 1996; Ullman and Gopnik, 1999), and syntax (Thordardottir and Weismer, 2002; Riches et al., 2010). It is important to note that the focus of this section is not on childhood motor speech disorders such as apraxia of speech, speech sound disorders, or articulation disorders. These subgroups of speech disorders are clearly linked to errors in motor programming and sometimes occur comorbidly with language impairment, but for the scope of the current discussion, the focus will remain on specific language impairment (also popularly referred to as primary language impairment—PLI). This section will extend the previous discussion of language being represented in motor, sensory and emotional areas of the brain to the interesting case of children with SLI who represent what happens when something goes wrong in language development. Perhaps this introduction is a bit misleading since I will spend a good portion of this section suggesting that what is frequently called *specific* language impairment may not be so specific to the language system after all.

An overview of how cognitive, linguistic, and motor skills develop together in early years and their important cross-domain relationships will set the stage for the discussion of later problems with language impairment. For instance, Angrave and Glenberg (2007) found a linear relationship between the ability to accomplish an action and the ability to say the word related to that action (e.g., drink), with the speech emerging about a year after the action emerged. Thal and Tobias (1992) found that pointing behaviors at 9 months of age were positively correlated with receptive vocabulary skills. Similarly, Acredelo and Goodwyn (1988) found that the rate of expressive vocabulary acquisition is directly related with the earlier ability to label objects using gestures. Standardized language scores at 22 months of age are also predicted by the ability to demonstrate appropriate and functional use of toys during play at 13 months (Ungerer and Sigman, 1984). These studies provide evidence that fits in well with the type of language acquisition model proposed by Glenberg and Gallese (2012) that emphasizes the importance of action-based language.

The relationship between motor and linguistic development is not unidirectional. A series of studies by Green et al. (2000, 2002) showed that important changes in lip and jaw movements occur at ~2 years of age, at the same time as the famed “vocabulary growth spurt” occurs. They found that the production of bilabials changed from being a primarily jaw-driven motor act to involving both the jaw and the lips (Green et al., 2000). The authors propose that these changes in motor development are brought about due to increasing demands of the linguistic and cognitive systems. Even in adults, when speech motor stability is measured, researchers have found that stability is directly related to the syntactic complexity of the stimuli (Walsh and Smith, 2002; Kleinow and Smith, 2006), that stability increases with age (Goffman and Smith, 1999; Walsh and Smith, 2002), and that second language learners' speech motor stability increases with second language proficiency (Nip and Blumenfeld, 2015). Finally, Nip et al. (2011) found that speed of jaw opening and jaw range of motion were both significantly and positively correlated with the perception and concepts subtest of the Batelle Developmental Inventories, Second Edition (BDI-2, Newborg, 2005) which assesses an infant's active sensorimotor interactions with the immediate environment. As would be predicted by embodiment theory, research in this area demonstrates tight links between speech motor and language abilities (perhaps because these are theorized to be two parts of the same neural network) across the developmental continuum.

Based on the preceding evidence, one might predict that children who have later language impairment would also demonstrate motor deficits. In 2001, Hill published a meta-analysis of previous work done with children with SLI and concluded that this impairment is frequently associated with deficits in both fine and gross motor skills, so much so that it should be considered the rule rather than the exception. While her review included children with a variety of speech and language disorders, only those that involved children with SLI will be reviewed here.

Several studies found that children with SLI demonstrated fine motor deficits using a task that required them to move small pegs from one location to another in a timed fashion (Bishop and Edmundson, 1987; Powell and Bishop, 1992). Children with SLI moved fewer pegs in the time allotted compared to a control group. Children with SLI have also been shown to demonstrate relative difficulty with a finger opposition task, which requires children to touch each of their fingers to their thumb as fast as they can while both accuracy and time are tracked (Johnston et al., 1981; Stark and Tallal, 1981; Katz et al., 1992). Children with SLI were both slower and less accurate at completing this task. Repetitive tapping and threading beads onto a string are other areas in which children with SLI have been shown to be slower than matched controls (Hughes and Sussman, 1983; Powell and Bishop, 1992). In terms of gross motor control, children with SLI primarily demonstrate balance issues as demonstrated by their diminished ability to hop or stand on one foot (Johnston et al., 1981; Stark and Tallal, 1981; Powell and Bishop, 1992).

An alternative methodology that researchers have undertaken to explore the comorbidity of SLI and motor deficits is by comparing children with SLI to children with developmental coordination disorder (DCD). DCD is typically diagnosed using a standardized test of motor abilities, such as the Movement Assessment Battery for Children (Movement ABC, Henderson and Sugden, 1992), and the diagnosis is given to children who fall below the 15th percentile compared to age-matched peers (Hill, 2001). Studies that previously used the Movement ABC found that between 60 and 90% of children with SLI performed below the 15th percentile on this test (Robinson, 1991; Hill, 1998; Hill et al., 1998; Rintala et al., 1998; but note that the Robinson study involved children with concomitant speech *and* language disorders). Therefore, the majority of children in these studies qualified for a diagnosis of DCD, suggesting that there is a large overlap between deficits in language and motor abilities.

Hill (1997) compared a group of children with SLI to a group of children with DCD, a group of age-matched controls, and a group of younger controls on their performance on the finger opposition task and the repetitive tapping task. She found that children with SLI's performance on the finger opposition task was statistically different from age-matched controls but was similar to the younger control group and the group with DCD. However, on the repetitive tapping task, no group differences were found (in contrast to Hughes and Sussman, 1983). Powell and Bishop (1992) also compared children with SLI, DCD, and matched controls and found that children with SLI performed similarly to children with DCD on all motor tasks and tasks of visual discrimination, but not on tasks of visuo-spatial processing (SLI>DCD on these subtests). These findings led Hill (2001) to conjecture that co-occurring language and motor deficits may be indicative of a global neurodevelopmental delay or that there may be an “anatomical contiguity of the neural substrates subserving language and motor functions” (p. 165). It is exactly this second hypothesis that embodied theories of language would predict to be correct.

Several more recent studies have continued to investigate this intriguing relationship between language and motor skills. Archibald and Alloway (2008) investigated this relationship from the opposite viewpoint by looking at the language abilities of children diagnosed with DCD and demonstrated that these children, on average, exhibit expressive language difficulties, providing further evidence for overlap between the two skill sets. Flapper and Schoemaker (2013) and Finlay and McPhillips (2013) both demonstrated that ~30% of children with SLI qualify for a diagnosis of DCD (note that this number is lower than earlier studies reported by Hill, which may be due to a stricter definition of SLI in both of the above studies compared to Hill's earlier studies). Vukovic et al. (2010) further explored how leg coordination, arm coordination and ability to imitate arm and hand gestures related to expressive vocabulary, receptive vocabulary, and articulation. Besides the (now somewhat repetitive) finding that children with SLI performed significantly worse than control children on all tests of motor abilities/coordination, the authors found that imitation of complex arm and hand movements predicted expressive vocabulary in both typically developing and SLI groups while imitation of simple arm and hand movements predicted receptive vocabulary only in the SLI group. Additionally, coordination of arms was highly predictive of expressive and receptive vocabulary in the control group. Zelaznik and Goffman (2010) also report generally poorer performance of children with SLI on motor tasks, but note that, on tasks that required timing precision, there were no group differences, perhaps suggesting a relative strength in this area.

Although articulation and speech sound disorders do not fall under the umbrella of specific language impairment, research has demonstrated that children with SLI still demonstrate subclinical deficits in this area. Findings in this area included that speech rate was overall slower for children with SLI (Andrade et al., 2014) and that articulatory movements were less stable in children with SLI. Higher articulatory variability was correlated with poorer performance on fine motor skills (performed with the hands; DiDonato Brumbach and Goffman, 2014). Additionally, a recent study used gummy bears as bite blocks and had children with SLI and a control group repeat nonwords. They found that the bite block was more disruptive for children with SLI when they produced 2 or more syllable-words and theorized that this may be related to difficulties with motor planning. Interestingly, SLI is 1.5–3 times more likely to occur in boys than girls (Tomblin et al., 1997; Broomfield and Dodd, 2004) and Smith and Zelaznik (2004) found that boys (until age 5 years) show a slower maturational course of speech motor development. Evidence that has accumulated over the past two decades continues to point to pervasive deficits in motor skills among children with SLI.

One area of motor ability that may be particularly relevant to theories of embodied cognition is that of hand gestures, since Broca's area is responsible for both articulation and hand movement, and authors have implicated neural exploitation of this area for both speech and action control. Based on this theory and the previous evidence presented here, one would predict that children with SLI would exhibit difficulties in producing communicative gestures. Hill reviewed a group of studies concerning children with SLI and their ability to perform transitive gestures (e.g., show me how you brush your teeth) and intransitive gestures (e.g., show me how you blow a kiss) as well as their ability to imitate novel hand shapes. The overwhelming message of these studies was that children with SLI had difficulty with production and imitation of both types of meaningful gestures and with imitation of novel hand shapes (Dewey et al., 1988; Hill, 1998; Hill et al., 1998).

However, more recent studies call into question these previous findings. Botting et al. (2010) found no differences in the ability of children with SLI to produce representational gestures. In fact, Iverson and Braddock (2011) found that children with SLI were more likely to produce gestures and that the frequency of gestures was negatively correlated with language abilities (i.e., the worse the language skills, the more gestures produced). Evans et al. (2001) found that children with SLI were more likely to use gestures to convey information complementary to the information conveyed in speech, which the authors suggest may be an attempt to externalize some of the processing load to reduce demands on a less stable system of phonological representation. Both sets of studies (the earlier studies reviewed by Hill and the more recent studies reported here) involved children of similar ages, suggesting that differences in task difficulty and individual differences may be the reason such different results emerged. More research into the relationship between motor, linguistic, and gesture abilities are necessary to fully elucidate how these abilities are related, especially since researchers have demonstrated that gesture may help children ground knowledge in sensorimotor experience (Beilock and Goldin-Meadow, 2010).

Based on this evidence that children with SLI exhibit both fine and gross motor subclinical deficits, combined with the evidence from researchers studying the embodied nature of language, informed hypotheses can be formulated to extend the current theoretical approach and describe the difficulties that children with SLI have. If there are weaknesses and instabilities in speech motor control, even at a subclinical level, in children with SLI, then the neural network that connects acoustic input, articulatory motor plans and semantics may be disrupted. Many investigators have theorized that deficits in phonological working memory or instability of phonological representations are at the core of the difficulties that children with SLI experience (Montgomery, 1995; Adams, 1996; Weismer et al., 1999; Evans et al., 2001). However, as embodied theories would predict, at least some part of phonological representation is represented in motor cortex. Therefore, overlapping deficits could be conceptualized to be due to overlapping responsibilities of the motor cortex in both action control and language processing. In fact, researchers have found that the pars triangularis (one part of Broca's area) is significantly smaller in children with SLI and the severity of the language impairment was correlated with the degree of this abnormality (Gauger et al., 1997). This finding provides neurostructural evidence for the critical role of motor control in language pathologies.

According to Glenberg and Gallese's (2012) *ABL* (Action-based language) model, based on the HMOSAIC account of motor control, performing an action should prime articulation of the word that labels that action (and vice versa); so one testable hypothesis that may be generated from this theory is whether a child who has difficulty performing an action also has a weaker or nonexistent prime of the related word or sentence. Similarly, if mirror neurons respond similarly when performing an action as when observing an action, it follows that children who have difficulty performing actions may also have difficulty in perceiving them. Such perceptual deficits may lead to further difficulties in acquiring new vocabulary since children may have difficulty learning appropriate ways to interact with their environment (affordances), and therefore have slowed or interrupted development of modules for language and action control. Finally, if language comprehension occurs through simulation of the actions, perceptions, and emotions described, someone who has motor deficits may specifically struggle to understand action verbs, which is a hallmark of SLI, at least in English (Conti-Ramsden and Jones, 1997).

At the very least, the results and theories reported here suggest that motor abilities should be assessed in the process of diagnosis of children with SLI (and language abilities should be assessed when diagnosing DCD). However, there is also the possibility that novel interventions may be developed based on the principles of embodied cognition. Developing language interventions that emphasize that the goal of understanding language is to guide appropriate action may be particularly beneficial and may further the theoretical approach. Therefore, embedding language intervention into activities that require the child to follow a direction and perform the actions required by the direction may prove to have positive results. Activities that require children to get their bodies involved in learning are also recommended (McClelland et al., 2015). Previous research has demonstrated that embodied learning can improve outcomes in areas that range from narrative text comprehension to mathematical equivalence to physics concepts and organic chemistry (Goldin-Meadow et al., 2009; Glenberg, 2011).

Similarly to what was argued about effective second language teaching, interventions designed for children with SLI should require children to interact with their immediate environment. For example, if the activity were to focus on proper use of verbs with the –ing morpheme, children would be asked to perform actions and then describe those actions as they perform them. Principles of Hebbian learning allow for development of testable hypotheses such as the idea that saying the correct form of the word (and necessarily also hearing it) while in the process of actually performing the action should strengthen the action-perception links that underlie language and ground concepts. The implications extend beyond speech and language therapy. It may also be possible that more children who are diagnosed with SLI should be referred for services with physical and occupational therapists so as to support simultaneous development of their motor and language skills. However, it should be noted that these systems (motor, perceptual, and linguistic) are not considered independent systems that should be focused on separately; instead it is recommended that speech therapists include action-based activities in their therapy activities and that physical and occupational therapists include language activities in their therapy activities so as to support development of neural networks that use motor, perceptual, and linguistic capacities.

## Author contributions

The author confirms being the sole contributor of this work and approved it for publication.

## Funding

The author is a doctoral student funded by NSF grant #1324807 titled EMBRACEing English Language Learners with Technology that focuses on using the theory of embodied cognition to develop educational technology to improve reading comprehension in elementary school children who are learning English as a second language.

### Conflict of interest statement

The author declares that the research was conducted in the absence of any commercial or financial relationships that could be construed as a potential conflict of interest.
